# Performance of Young Children on ‘‘Traveling Salesperson’’ Navigation Tasks Presented on a Touch Screen

**DOI:** 10.1371/journal.pone.0115292

**Published:** 2014-12-18

**Authors:** Hiromitsu Miyata, Shigeru Watanabe, Yasuyo Minagawa

**Affiliations:** 1 Japan Society for the Promotion of Science, Tokyo, Japan; 2 Graduate School of Human Relations, Keio University, Tokyo, Japan; 3 Faculty of Letters, Keio University, Tokyo, Japan; Brock University, Canada

## Abstract

**Background:**

The traveling salesperson problem (TSP) refers to a task in which one finds the shortest path when traveling through multiple spatially distributed points. Little is known about the developmental course of the strategies used to solve TSPs. The present study examined young children's performance and route selection strategies in one-way TSPs using a city-block metric. A touch screen-based navigation task was applied.

**Methodology/Principal Findings:**

Children (39–70 months) and adults (21–35 years) made serial responses on a touch screen to move a picture of a dog (the target) to two or three identical pictures of a bone (the goals). For all the versions of the tasks, significant improvement in measures of performance was observed from younger to older participants. In TSPs in which a specific route selection strategy such as the nearest-neighbor strategy minimized the total traveling distance, older participants used that strategy more frequently than younger ones. By contrast, in TSPs in which multiple strategies equally led to the minimal traveling distance, children tended to use strategies different from those used by adults, such as traveling straight to the farthest goal first.

**Conclusions/Significance:**

The results primarily suggest development of efficient route selection strategies that can optimize total numbers of movements and/or solution time. Unlike adults, children sometimes prioritized other strategies such as traveling straight ahead until being forced to change directions. This may reflect the fact that children were either less attentive to the task or less efficient at perceiving the overall shape of the problem and/or the relative distance from the starting location to each goal.

## Introduction

The traveling salesperson problem (TSP) is a problem in which a traveler is required to find the shortest possible path when visiting multiple locations once before returning to a starting point. It is a renowned optimization problem that researchers on mathematics, computer science, and artificial intelligence have intensively addressed over the past decades (for overviews, see [1]–[3]). The TSP is computationally a difficult problem, and multiple heuristics and approximation algorithms have been proposed [4]. Less frequently, empirical studies on experimental psychology and cognitive science have addressed the mental mechanisms that humans may use when solving TSPs [5]–[11]. These studies concur in finding that adult humans quickly attain close-to-optimal solutions in TSPs with varying numbers of visiting locations. For classic paper-based TSPs that require returning to the starting location, models developed by McGregor and colleagues assume that adults rapidly perceive the convex hull of the problem when encountering TSPs having 10 to 100 nodes. They suggest that such low-level perceptual processes can produce high-quality TSP solutions [9], [10], [12]. In addition, higher-order and more analytic processing may also mediate adult humans' performance on these TSPs [13].

To date, little empirical research has addressed the development of the mental strategies used to solve TSPs. Van Rooij et al. [14] examined the performance of 7-year-old and 12-year-old children and adults on paper-based TSPs with 5, 10, and 15 visiting points. 7-year-old children frequently selected routes that were either optimal or close to optimal. Systematic improvements in performance with age were also observed, with older participants selecting routes with shorter traveling distance than younger ones. Using a real-world setting in a room similar to the TSP, Pellicano et al. [15] instructed 8- to 14-year-old typically developing and autistic children to search an array of 16 locations to find a hidden target. Typically developing children followed the optimal and systematic search paths to a greater extent than autistic children. More accumulation of systematic empirical studies involving younger children should be required in this frontier, because the capacity to use efficient solution strategies in situations similar to TSPs should be beneficial during the navigation behavior of preschoolers in everyday life. Considering the substantial development of spatial and motor skills during these ages [16], [17], it would be natural to assume that development of efficient performance would occur in older preschoolers in terms of measures of performance such as solution time and number of responses required to complete the task.

We examined the solutions of young children between 3 and 5 years of age on TSPs presented on a touch screen. We applied a navigation task that had previously been used to examine pigeons' (*Columba livia*) performance on maze problems and TSPs and planning. Consequently, the present TSPs used a city-block metric instead of a Euclidean metric. Miyata et al. [18] developed the original version of the task in which pigeons made a chain of pecking responses on an LCD screen to move a red square (the target) to the location of a blue square (the goal). The birds determined the direction of each movement by pecking at one of the four small dots (the guides) that appeared around the target. Using this navigation paradigm, pigeons appeared to plan ahead before starting to make a detour around the barrier [18], and to plan one future step both during and before solution of the maze task [19], [20]. Miyata et al. [16] applied some of these maze tasks to 3- to 4-year-old human children, which revealed more efficient capacities for planning and inhibition/reengagement in older participants (see also [21]). These computer-assisted tasks are beneficial compared to paper-based tasks because they can systematically record details of participants' behavior in automatic ways. This seems especially relevant considering the fact that evidence on young children's spatial behavior in touch screen-based settings still remains scarce [16].

The present study is most directly based on Miyata and Fujita [22], in which pigeons solved computerized one-way TSPs by moving the target to two or three identical goals. The birds had no need to revisit the starting location. In all four different variations of these TSPs, the pigeons frequently selected the nearest goal as the initial goal to visit. These data suggested that one major strategy that the birds may have used was the nearest-neighbor strategy, or the rule to select the next nearest point at each given location. Further, we also found that pigeons frequently made round trips when the three goals and the target starting location held each corner of a square. The pigeons also exhibited tendencies to initially visit the cluster of two goals rather than the other isolated goal (see also [23]). These data provided consistent and additional evidence on TSP performance by pigeons ([24], see also [25]) as well as by nonhuman primates [26]–[27].

In addition to the aforementioned theoretical models, studies involving humans as well as nonhuman animals have suggested use of various strategies or heuristics when solving one-way TSPs. The present study introduced different versions of the TSPs having two to three goals in order to examine the following strategies. We generally expected to find use of more efficient or closer-to-optimal route selection strategies in older participants. The first strategy we examined is the nearest-neighbor strategy, which humans as well as pigeons are suggested to use frequently [22]–[24], [28]. Because the nearest-neighbor strategy is the optimal one at least in TSPs having two goals, this strategy may well be more frequent in older participants for these problems.

The second heuristic is the strategies potentially used when the three goals and the starting location hold each corner of a diamond. When the distance between the two goals nearer from the starting location is sufficiently short, the optimal route involves crossing the intersection of the diamond. Gallistel and Cramer [27] showed that vervet monkeys (*Chlorocebus aethiops*) in the wild are efficient at using this strategy. Older participants would be expected to use this strategy more frequently than younger ones. By contrast, when the distance between these goals is longer so that the goals and the starting location hold each corner of a square, the round trip following the perimeter of the diamond is just as efficient as the crossing route in terms of the total traveling distance in a city-block metric. Thus, developmental changes may not be observed in these cases. In addition, it is also possible to travel straight ahead to visit the farthest goal first until being forced to change moving directions. Even though this strategy does not minimize the total traveling distance, participants may possibly use it if they fail to accurately perceive the relative distance from the starting location to each goal.

Third, one variation of the former diamond-shaped configuration involves the cases in which three goals are equidistantly located along a straight line [22]. In these cases, both making a round trip and traveling straight ahead to first visit the goal in the middle (this also matches the nearest-neighbor strategy) are possible, although these strategies make no difference in terms of minimizing the traveling distance. With lack of a single optimal strategy, distinctive developmental changes in strategies used may be unlikely to be observed. Nevertheless, younger and older participants might show different behavioral tendencies by, for example, continuing to use the strategies that they once selected in the former diamond-shaped problems.

Fourth, we examined the clustering strategy when the two goals are adjacent to each other while the other is isolated. For adult humans, the clustering strategy assumes that multiple goals are first clustered and that those clusters are then ordered using other heuristics such as the nearest-neighbor strategy [28]. Studies involving nonhuman animals concur in finding that they prefer to initially travel to the larger clusters of nodes before visiting the remaining nodes [24], [27], [29]. This preference seems to have adaptive value because the strategy corresponds to quickly obtaining more resource intake before it is possibly taken away [22]. Human children and adults may also show comparable preferences for the clusters, assuming that these participants are sensitive to the temporal order of obtaining a larger amount of reward.

In the present study, we examined the developmental course of performance in preschool children and adults when solving touch screen-based multi-goal navigation tasks that represented TSPs. Participants solved different versions of one-way TSPs having two or three goals by making serial responses using their fingers. We used materials for the navigation task that were previously applied for young children [16]. Configurations of the stimuli used in each TSP version were modified based on those previously applied for pigeons [22], which allowed for the possible strategies and predictions mentioned above.

## Materials and Methods

### Participants

Twenty-nine healthy Japanese children (12 girls and 17 boys; age, 39–70 months; mean age  = 52.1 months, *SD*  = 8.5 months) and 12 healthy Japanese adults (8 females and 4 males; age, 21–35 years; mean age  = 24.9 years, *SD*  = 3.6 years) participated. Two of these participants (male, 51 months; male, 54 months) refused to cooperate after completing Test 2 and Test 3, respectively. For another four participants (male, 48 months; female, 22 years; female, 23 years; female, 28 years) only 2 trials (one straight-small and the other straight-large) were run during Test 3, because these participants were recruited at the beginning of the testing period when the number of trials was not fixed. These missing data were excluded from analysis. Three other children (female, 36 months; male, 36 months; female, 42 months) refused to cooperate during instructions and thus were not included in the analysis. Children were divided into the younger (*N* = 14; 5 girls and 9 boys; age, 39–51 months; mean age  = 45.1 months, *SD*  = 3.9 months) and the older (*N*  = 15; 7 girls and 8 boys; age, 52–70 months; mean age  = 58.7 months, *SD*  = 6.0 months) groups by making each group size equivalent for the analysis. To recruit the children, we used our own advertisement that solicited cooperation for developmental studies at either behavioral or neuronal levels. Written informed consent was provided by all children's caretakers and by all adult participants when they agreed to cooperate. A small monetary reward was given to all children and their caretakers after the experiment. The Ethics Committee of Keio University approved the study (Approval No. 09037-3).

### Settings

A 46-cm (18.1 inches) TFT LCD monitor with a built-in Ultrasonic Surface-Wave touch screen (AS4641D, Iiyama, Tokyo, Japan) was used to present the tasks, which was located on a rectangular table (60 cm wide ×45 cm long ×31 cm high). When the participants preferred to perform the tasks while sitting, a small chair (16 cm high) was placed in front of the monitor. A desktop personal computer (Dell Precision 650; CPU: Intel, Xeon^TM^) placed outside the experimental room controlled presentation of stimuli on the monitor and recording of the participants' responses. The experimenter wrote the program in Microsoft VisualBasic 6.0.

### Materials

We used a set of computer-generated visual stimuli involving a target, arrows named *guides*, goal(s), and an outer frame. All these components of stimuli were the same as those used in Miyata et al. [16], and were combined to construct a TSP. The target was a white square within which there was a picture of a dog (50×50 pixels, about 13×13 mm). Four blue arrows (34×32 pixels, about 9×8 mm) could appear around the target as guides. The picture of the dog was created by modifying a downloaded copyright-free illustration. The goal was an original illustration of a bone and fitted within a square area of 50×50 pixels. Two or three identical goals were used in each test trial. The whole task appeared within a square-shaped area (550×550 pixels, about 14.5×14.5 cm) having a 9×9 matrix, of which 10-pixel-wide blue lines defined the outer frame.

### Procedure

The study was conducted in a quiet experimental room specially arranged to accommodate participants. This section first describes the procedure for the children who participated. After arrival at the location, the participant and the caretaker were guided to the experimental room. The experimenter (HM) and the assistant(s) played with the visitors, usually using toys equipped in the room, until they established a rapport with the participant. During this period, the experimenter explained the content of the study to the caretaker and obtained informed consent. During the experimental session, the participant sat in front of the table, either on the floor or on the chair. The experimenter sat on either the right or left side of the participant and gave instructions or encouragement when necessary. The caretaker and the assistant(s) either sat close to the participant and the experimenter or waited outside the experimental room. Each session consisted of an instruction phase (Practice) and the subsequent four test phases (Tests 1–4). A short resting period was given after Practice, Tests 1, and Test 2 when the participant wanted it. The same general procedure as in the previous phase was replicated during the next phases, but with the changes described below. The procedure for the adults was identical to that for the children, except that there was no rapport phase or resting period and adults were not provided with any additional help or encouragement.

#### Navigation task

**Figure 1:**
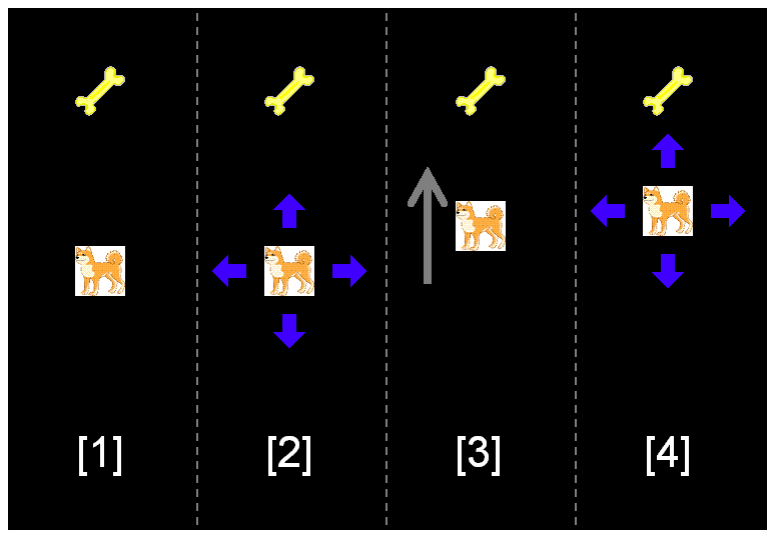
Illustration of the navigation task used in order to solve the TSPs. The white square having a picture of a dog represents the target, and the picture of a bone at the top represents the goal. The gray arrow depicted in [3] represents the target's movement. See the Procedure section for description of each stage of navigation ([1]–[4]).

Throughout the experiment, we used the same navigation task as in Miyata et al. [16], which had originally been developed for pigeons [18]–[20], [22], [23]. The task was to move a picture of the dog (the target) to a picture of a bone (the goal) by making serial finger-touches on the screen (**Fig. 1**). At the beginning of the problem, a touch on the target resulted in four guides (arrows) at the four positions–above, below, right, and left–surrounding the target (**Figs. 1** **(1) and (2)**). Touching one of the guides immediately caused all four guides to vanish and resulted in the target moving in the direction of the responded guide in animation (**Fig. 1** **(3)**). The target moved 60 pixels (about 15 mm) in 0.6 s, after which the four guides reappeared (**Fig. 1** **(4)**). Any responses to the monitor while the target was moving caused no outcome; thus each touch was discrete and participants did not need to keep touching the screen with their fingers. In this way, participants were able to move the target freely within the frame. Then, the task of the participants was to move the target as many times as necessary until the target arrived at the location of the goal.

#### Practice

The experiment started with an instruction to perform the navigation task. First, the experimenter verbally instructed the participant to lead a hungry dog to the location of the bone as quickly as possible. There was no explicit instruction to minimize total traveling distance. Then a finger-touch on the picture of the dog depicted as a self-start key resulted in a blank display of variable duration ranging from 1 to 3 s. Any touches on the monitor had no effect on the display during this interval, which was followed by a stimulus display. The target initially appeared at the center of the framed area, and a single goal appeared at one of the 12 locations three movements away from the target starting location. A successful navigation of the target to the goal caused the stimulus display to disappear, immediately followed by a chime sound (2 s) and by a display of the happy dog with one bone and one star. To facilitate quicker solution of the problem, there was a slant (or proportional decline) in the duration of this visual reward, so that solution in shorter time was followed by a longer reward display. Specifically, solutions in 15 s, 30 s, and 45 s each corresponded to the subsequent reward displays of 4 s, 3 s, and 2 s. Then, there was a 3-s blank display until the next trial started. There was no limit to the duration of each trial, and the experimenter or the caretaker aided the participant's performance by showing examples of movements or a whole trial when necessary. This phase was run for 3 trials.

#### Test 1: two goals

**Figure 2:**
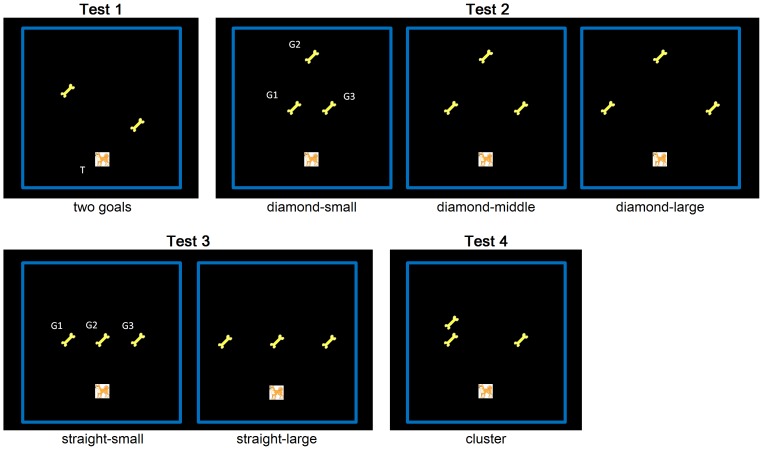
TSP versions used during each test phase (Tests 1–4). The picture of the dog at the bottom of each task (T) represents the starting location of the target, and the pictures of the bone (G1, G2, [and G3]) represent the goals. Variations in the target's starting location are available by rotating each figure by 90, 180, or 270 degrees.

In the first test phase following Practice, a one-way TSP having two goals was introduced (**Fig. 2**). There were four possible starting locations of the target, i.e., above, below, right, and left, each at three movements away from the center of the framed area. As illustrated in **Fig. 2**, the smallest numbers of movements from the starting location to the nearer and the farther goals were 4 and 6, respectively. Participants could select either sequence of goals to travel, i.e., nearer goal first or farther goal first, with no need to revisit the starting location. The reward display of the dog with two bones and two stars appeared immediately after arriving at the second goal. Reaching the first goal in each trial extinguished the goal from the display and resulted in a chime sound (0.4 s) different from that played after visiting the final goal. During all test phases, the experimenter, the caretaker, or the assistant(s) did not do anything other than verbally encouraging the participants without aiding the participants' performance. Throughout the tests, participants were not told which specific routes they should select. There were 4 (target starting location) ×2 (configurations of the nearer/farther goal)  = 8 variations of this task. The test was run for 2 trials, during which two of these variations were presented in a pseudo-randomized order.

#### Test 2: three goals–diamond

**Figure 3:**
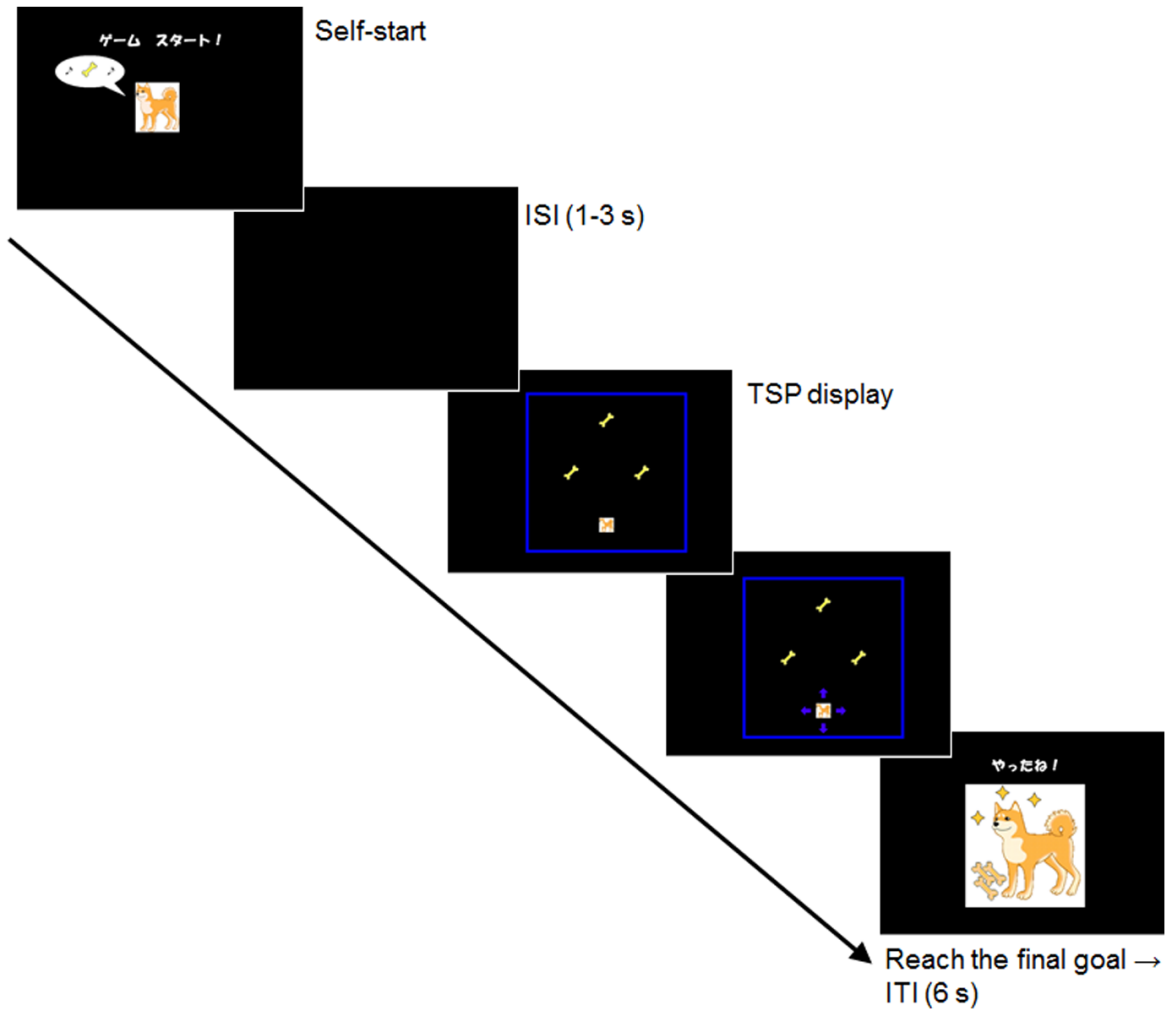
Diagram showing a typical example of a trial during the test. ISI  =  interstimulus interval; ITI  =  intertrial interval. The Japanese characters on the self-start display mean “Start the game!” and those on the ITI display mean “You have got it!”.

(See **Fig. 3** for a diagram of a trial). Three goals, each named G1, G2, and G3 and numbered in a clockwise order, appeared within the outer frame so that these goals and the target starting location held each corner of a diamond (**Figs. 2** **and** **3**). As illustrated in **Fig. 2**, G2 was placed 6 movements away from the target's starting location along a straight line. G1 and G3 were placed at symmetrical locations and were 2, 4, or 6 movements apart from each other. These three versions of the diamond are referred to as *diamond-small*, *diamond-middle*, and *diamond-large*, respectively. Participants were allowed to select any of 6 possible sequences of goals to travel (G1→G2→G3, G2→G3→G1, etc.). Visiting the first and the second goals during each trial immediately resulted in the same shorter chime sound as in Test 1. Arriving at the final goal was immediately followed by the display of the happy dog with three bones and three stars (**Fig. 3**) and a longer chime sound. Each version (diamond-small, diamond-middle, and diamond-large) had 4 variations (target starting locations); two of these variations were each presented in a pseudo-randomized order during the 6 consecutive test trials.

#### Test 3: three goals–straight

Three goals, named G1, G2, G3, were placed numerically along a horizontal/vertical straight line. G2 was positioned at the center of the framed area and was three movements away from the starting location of the target. G1 and G3 both appeared along a line that is vertical to the line connecting the target starting location and G2. These two goals were located equidistantly, either 2 or 3 movements apart from G2. These different versions are referred to as *straight-small* and s*traight-large*, respectively (**Fig. 2**). Each version had 4 variations (target starting locations), and two of these variations were each presented in a pseudo-randomized order during the 4 consecutive test trials.

#### Test 4: three goals–cluster

Three goals were positioned so that two of them (either G1 and G2, or G2 and G3) were adjacent to each other to form a cluster while the other goal was isolated (**Fig. 2**). The nearer member of the cluster and the isolated goal were placed at symmetrical locations and were 5 movements away from the starting location of the target. The farther member of the cluster was next to the nearer toward the opposite side of the framed area from the target starting location. There were 2 (side of the cluster) ×4 (target starting location)  = 8 variations in total. This test lasted for 4 trials, during which four of these variations were presented once in a pseudo-randomized order.

## Results

Younger and older children and adults were analyzed as separate groups of participants. Tests 2 and 3 had different TSP versions with varying distances between G1 and G3. To clarify the differences between these versions, each version was analyzed separately. Accordingly, there were seven TSP versions in total. Based on previous studies [16], [22], we examined three measures of performance for each TSP version. The first was the mean number of the target's movements until arrival at the final goal in each trial. The second was task-solving time, defined as the median time from the moment the participants first responded to the target to the moment the target arrived at the final goal. The third was first response latency, or median time from the moment the TSP display appeared to the moment the participants first touched the target to make the guides appear in each trial. Data for these measures are summarized in **Table 1**. Number of movements, task-solving time, and first-response latency all decreased from younger to older children and from older children to adults for all TSP versions, with only two exceptions (number of movements for cluster and first-response latency for diamond-large).

**Table 1:** Measures of performance on each TSP version.

Measure	TSP	Younger children	Older children	Adults
N movements	Two goals	14.1 (5.3)	10.9 (1.3)))	10.0 (0.0)
	Diamond-small	12.1 (1.6)))	10.5 (0.6)	10.1 (0.3)
	Diamond-middle	16.0 (1.3)	14.9 (0.5)	14.3 (0.3)
	Diamond-large	20.0 (1.4)	18.9 (1.1)	18.0 (0.0)
	Straight-small	9.7 (0.9)	9.4 (1.1)	9.0 (0.0)
	Straight-large	13.2 (1.6)	13.1 (1.4)	12.0 (0.0)
	Cluster	11.1 (0.8)	11.1 (1.5)	10.2 (0.3)
Task-solving time	Two goals	29.3 (31.3)	23.3 (9.5)	11.6 (1.5)
(sec)	Diamond-small	24.1 (6.6)	15.1 (2.9)	10.9 (1.1)
	Diamond-middle	28.6 (6.1)	23.7 (4.0)	14.9 (1.9)
	Diamond-large	38.3 (7.1)	25.8 (5.0)	19.0 (2.4)
	Straight-small	18.1 (7.1)	13.3 (3.0)	9.2 (0.9)
	Straight-large	22.6 (6.1)	17.7 (2.7)	12.4 (1.7)
	Cluster	20.6 (4.5)	16.5 (3.3)	10.6 (1.2)
First-response	Two goals	4.0 (3.8)	3.0 (3.1)	1.4 (0.7)
latency (sec)	Diamond-small	3.3 (2.6)	2.4 (1.0)	1.1 (0.5)
	Diamond-middle	3.6 (1.6)	2.5 (3.1)	1.2 (0.5)
	Diamond-large	2.3 (1.4)	2.4 (0.9)	1.5 (1.1)
	Straight-small	2.9 (2.2)	2.2 (1.5)	1.3 (0.4)
	Straight-large	2.3 (13.2)	1.8 (2.6)	1.4 (0.6)
	Cluster	2.2 (1.8)	1.7 (0.7)	1.2 (0.5)

Mean numbers of the target's movements, median task-solving time, and median first-response latency are shown for each group. Standard deviations are shown in parentheses.

For each TSP version and measure of performance, a one-way analysis of variance (ANOVA) with group as a between-participants factor was used for comparisons between the three groups. These ANOVA results are summarized in **Table 2**. The main effect of group was significant for all TSP versions and measures except for two cases in number of movements and two cases in first-response latency. Multiple comparisons with Bonferroni correction revealed that, for all TSP versions, older children and adults showed better performance than younger children either in task-solving time or in number of movements. There were also cases in which older children showed performance equal to adults in these two measures. Multiple comparisons for first-response latency generally showed trends parallel to task-solving time and number of movements, although statistical outcomes were less apparent for this measure. These data show more efficient performance on the TSPs by older participants, most apparently in solution speed but also in the number of manipulations. In addition, quickness to make the initial response after the task display appeared also tended to be improved from younger to older participants.

**Table 2:** Results of one-way ANOVA for each TSP version and measure of performance.

					Multiple comparisons (Bonferroni corrected)		
Measure	TSP	Main effect: group			Younger children vs. Older children	Younger children vs. Adults	Older childrenvs. Adults
		*df*	*F*	*p*	*p*	*p*	*p*
N movements	Two goals	2, 38	5.881	0.006**	**0.034** *	0.009**	>0.999
	Diamond-small	2, 38	14.538	<0.001***	<0.001***	<0.001***	>0.999
	Diamond-middle	2, 38	13.094	<0.001***	0.006**	<0.001***	0.181
	Diamond-large	2, 38	10.913	<0.001***	**0.025** *	<0.001***	0.145
	Straight-small	2, 37	1.960	0.155	-	-	-
	Straight-large	2, 37	3.057	0.059	-	-	-
	Cluster	2, 36	3.308	**0.048** *	>0.999	0.099	0.086
Task-solving time	Two goals	2, 38	6.431	0.004**	0.157	0.003**	0.287
(sec)	Diamond-small	2, 38	33.356	<0.001***	<0.001***	<0.001***	0.0099**
	Diamond-middle	2, 38	32.672	<0.001***	0.004**	<0.001***	<0.001***
	Diamond-large	2, 38	35.402	<0.001***	<0.001***	<0.001***	<0.001***
	Straight-small	2, 37	19.985	<0.001***	<0.001***	<0.001***	0.117
	Straight-large	2, 37	22.941	<0.001***	<0.001***	<0.001***	**0.013** *
	Cluster	2, 36	32.242	<0.001***	<0.001***	<0.001***	<0.001***
First-response latency	Two goals	2, 38	5.593	0.007**	>0.999	0.009**	**0.038** *
(sec)	Diamond-small	2, 38	10.387	<0.001***	**0.020** *	<0.001***	0.237
	Diamond-middle	2, 38	3.860	**0.030** *	>0.999	0.073	**0.047** *
	Diamond-large	2, 38	2.179	0.127	-	-	-
	Straight-small	2, 37	5.875	0.006**	0.539	0.005**	0.107
	Straight-large	2, 37	1.405	0.258	-	-	-
	Cluster	2, 36	4.258	**0.022** *	0.272	**0.019** *	0.660

*: *p*<0.05; **: *p*<0.01; ***: *p*<0.001.

To determine the routes each group of participants selected, we categorized the possible route selection strategies for each test phase. **Table 3** summarizes these strategies and the smallest numbers of movements for each case. For each group, TSP version, and route selection strategy, the number (and corresponding percentage) of trials selected was summed across participants and is indicated in **Table 3**. For each block of TSP version and group of participants, a chi-square goodness-of-fit test was conducted in order to compare the observed numbers of trials to the theoretical expectation that selection of each route is equal in frequency. Bonferroni correction was made for each TSP version. Results of these tests are summarized in **Table 4**. For Test 1, the routes were determined according to whether the nearer or farther of the two goals was visited first. The minimum number of movements before solution was two times smaller when selecting the *nearer first* route than when selecting the *farther first* route. All three groups of participants tended to use the nearer first route more frequently than the farther first route. The trend was more apparent in older participants, with adults visiting the nearer goal first for all the test trials.

**Table 3:** Number and proportion of trials with selection of each route.

TSP	Route	Younger children		Older children		Adults	
		N trials	% trials	N trials	% trials	N trials	% trials
Two goals	Nearer first (10)	20	71.4	25	83.3	24	100.0
	Farther first (12)	8	28.6	5	16.7	0	0.0
Diamond-small	Crossing (10)	16	57.1	25	83.3	23	95.8
	Round (12)	6	21.4	2	6.7	1	4.2
	G2 first (12)	6	21.4	3	10.0	0	0.0
Diamond-middle	Crossing (14)	9	32.1	15	50.0	18	75.0
	Round (15)	6	21.4	7	23.3	6	25.0
	G2 first (15)	13	46.4	8	26.7	0	0.0
Diamond-large	Crossing (18)	11	39.3	8	26.7	8	33.3
	Round (18)	5	17.9	6	20.0	16	66.7
	G2 first (18)	12	42.9	16	53.3	0	0.0
Straight-small	Crossing (13)	0	0.0	0	0.0	0	0.0
	Round (9)	8	32.0	4	13.3	13	61.9
	G2 first (9)	17	68.0	26	86.7	8	38.1
Straight-large	Crossing (17)	0	0.0	0	0.0	0	0.0
	Round (12)	4	16.0	2	6.7	14	66.7
	G2 first (12)	21	84.0	28	93.3	7	33.3
Cluster	Isolated first (10)	34	65.4	37	66.1	40	83.3
	Cluster-nearer first (11)	16	30.8	17	30.4	8	16.7
	Cluster-farther first (11)	2	3.8	2	3.6	0	0.0

For each block of TSP version and group of participants, percentages of trials with selection of each route were calculated based on the corresponding numbers of trials. For each route, the smallest number of movements to solve the TSP is shown in parenthesis.

**Table 4:** Results of chi-square goodness-of-fit tests regarding number of trials with selection of each route.

TSP	Younger children			Older children			Adults		
	*df*	*χ^2^*	*p*	*df*	*χ^2^*	*p*	*df*	*χ^2^*	*p*
Two goals	1	5.143	0.070	1	13.333	<0.001***	1	24.000	<0.001***
Diamond-small	2	7.143	0.084	2	33.800	<0.001***	2	42.250	<0.001***
Diamond-middle	2	2.643	0.800	2	3.800	0.449	2	21.000	<0.001***
Diamond-large	2	3.071	0.646	2	5.600	0.182	2	16.000	0.001**
Straight-small	1	3.240	0.216	1	16.133	<0.001***	1	1.190	0.826
Straight-large	1	11.560	0.002**	1	22.533	<0.001***	1	2.333	0.380
Cluster	1	6.480	0.033*	1	7.407	0.019*	1	21.333	<0.001***

*Crossing* routes for straight-small and straight-large and *cluster-farther first* routes for cluster were excluded from these tests. *: *p* <0.05; **: *p* <0.01; ***: *p*<0.001.

In Test 2, the six possible traveling sequences were categorized into the following three route selection strategies. The *crossing* routes involved traveling across the diamond along the midsection (i.e., G1→G3→G2 or G3→G1→G2). The *round* routes were those traveling either clockwise or counterclockwise while following the perimeter of the diamond (i.e., G1→G2→G3 or G3→G2→G1). The *G2 first* routes included the remaining two cases in which the farthest goal was visited first (i.e., G2→G1→G3 or G2→G3→G1). For diamond-small and diamond-middle, the crossing routes matched the optimal strategy with the shortest traveling distance. The crossing routes were most frequently used in both these TSP versions (except for diamond-middle for younger children), with the trends becoming more apparent for older participants. For diamond-large, the three route selection strategies made no difference in terms of the total number of movements. Adults most frequently selected the round routes and never started by visiting G2. By contrast, children frequently visited G2 first and thereby selected each route equally often (**Tables 3** and **4**).

The same three categories of route selection strategies were applied for Test 3. Both the round and the G2 first routes equally minimized the number of movements, whereas the crossing route traversing from G1 to G3 (or vice versa) without visiting G2 was obviously less efficient. Because there were no trials in which the crossing routes were selected, the chi-square goodness-of-fit tests involved only the round and the G2 first routes (**Tables 3** and **4**). For both straight-small and straight-large, adults selected the round and G2 first routes equally often. In contrast, children frequently visited G2 first, with the trends more apparent for older than for younger children. These patterns are in line with those observed for diamond-large, showing a clear distinction between children and adults and the children's tendencies to start by visiting G2.

In Test 4, trials were divided according to which of the three goals was visited first, because our interest here focused on whether or not participants preferred to visit the cluster of multiple goals at the beginning stage of solution. *Isolated first*, *cluster-nearer first*, and *cluster-farther first* each refer to the cases in which the isolated goal, the nearer member of the cluster, and the farther member of the cluster were visited first. Isolated first was the optimal strategy to minimize the traveling distance, whereas the cluster-nearer first and cluster-farther first were equally less efficient because they required one more movement than isolated first. Because the cluster-farther first routes accounted for less than 4% of the trials (0–2 trials) for each group of participants, the chi-square tests involved only the isolated first and cluster-nearer first routes (**Tables 3** and **4**). Participants for all groups selected the isolated first routes more frequently than the cluster-nearer first routes, with the trends most apparent for adults. By contrast, both younger and older children selected the cluster-nearer first routes in around 30% of the trials (**Table 3**). Thus, neither children nor adults tended to prefer visiting the cluster of the goals first but instead they both frequently used the strategy that minimized the total traveling distance, although children appeared less likely to stick to the optimal strategy.

## Discussion

The present study examined performance of preschool children and adults on “traveling salesperson” navigation tasks having two or three goals on a touch screen. The TSPs were based on those in our previous study with pigeons using a city-block metric [22], and materials and general procedure were comparable to those previously used to examine young children's performance on maze tasks and planning [16]. Throughout the tested TSP versions, performance tended to improve from younger to older children and from older children to adults, with older children showing performance approaching that of the adults for some conditions. These trends were most apparent in terms of solution time but also in number of total movements and latency of the initial response. These general findings suggest development of efficient solution performance in older participants when coping with these computerized problems.

Analyses of the route selection strategies used revealed group differences and unique tendencies in each TSP version. For Test 1 using TSPs with two goals, the nearest-neighbor strategy to start by visiting the nearer goal also matched the optimal one. As predicted, proportions of trials in which this strategy was used tended to be higher toward older participants, with both older children and adults selecting this route more frequently than expected by chance. This would suggest development of efficient route selection strategies at least concerning this simplest version of the problems.

In Test 2, diamond-shaped configurations involving three goals and the target starting location were introduced. When the distances between G1 and G3 were either 2 or 4 movements (i.e., diamond-small, diamond-middle), the crossing route traveling across the midsection of the diamond was the most efficient to minimize the traveling distance. Consistent with predictions, this strategy tended to be more frequent in older participants, even though neither group of children showed statistically significant outcomes for diamond-middle. These data seem to support the notion that older participants were more efficient at optimizing the traveling distance than younger ones. When the distance between G1 and G3 was 6 movements (i.e., diamond-large), both the crossing and round routes were equally efficient in total traveling distance. Nevertheless, contrary to expectations, the data revealed different tendencies between children and adults. Children used different strategies equally often while adults most frequently made round trips. These trends appear to agree with the previous literature suggesting that adults tend to perform on TSPs based upon perception of the overall shape of the figure such as the convex hull [9], [10], [12], even though the numbers of nodes are small in the present study. Interestingly, there were cases in which both younger and older children first visited G2, or the goal farthest from the starting location, most apparently for diamond-large but also for the other diamonds. Such cases were never observed for adults. One possible reason for this trend would be that children failed to precisely measure the relative distance from the starting location to each goal. It might also be possible that children were less attentive to the task itself than adults. In either case, these trends to “start by traveling straight ahead” seem consistent with the notion that children are less sensitive to the sequence of goals to visit compared with adults.

Test 3 was a variation of Test 2, but G2 was located on a straight line connecting G1 and G3. Contrary to our expectations, route selection strategies largely differed between children and adults. Adults selected the round and the G2 first routes equally often, but children frequently started by visiting G2 (the nearest goal). This tendency to start with G2 was more apparent for older children but was consistent in younger children as well. These trends are generally consistent with those observed in Test 2 (especially in diamond-large), parallel to the notion that adults take into consideration the overall shape of the figure whereas children do not. Because visiting G2 first matched the nearest-neighbor strategy in Test 3, the children's behavior could also be interpreted as traveling straight ahead to the nearest location before changing directions.

For Test 4, our initial hypotheses assumed a clustering strategy and a preference for initially visiting the cluster for both children and adults. Contrary to these predictions, both adults and children more frequently started by visiting the isolated goal than by visiting the nearer member of the cluster. Because this isolated first strategy was the optimal one to minimize the total traveling distance in Test 4, these results seem to show participants' efficient route selection strategy and its development. In other words, the preferences for clusters as suggested in animal studies [24], [27], [29] were not observed. This may reflect the fact that numbers of nodes and distances between them were far smaller than those in real-world settings such as foraging situations, and that participants had little need to prioritize the visit to the clusters of nodes.

Taken together, various route selection strategies and developmental changes were observed for different TSP versions. For TSP versions in which one particular route selection strategy minimized the traveling distance (Test 1, diamond-small and diamond-middle in Test 2, and Test 4), older participants overall tended to use that strategy more frequently than younger ones. These seem consistent with the idea that older participants are more efficient at selecting routes that can optimize the total number of movements and/or solution time. In contrast, for other versions in which multiple route selection strategies made no difference in minimizing the total traveling distance (diamond-large in Test 2 and Test 3), adults and children showed different trends in route selection strategies. These differences support the notion that adults tend to act upon the overall shape of the figure, whereas young children may prioritize other strategies such as traveling straight ahead until being forced to change directions.

These interpretations still require cautious considerations. One point to note is that the developmental changes observed in the present study may reflect not only the difference in task performance including heuristics but also difference in understanding of the task requirements. Specifically, considering the age of the younger children, it appears plausible that these participants were poorer at understanding the requirements of the task compared with the older children or the adults. For example, a verbal instruction given to all participants in this study was to move a hungry dog to the location(s) of the bone(s) as quickly as possible. Given this instruction, adults should understand that the desired optimal strategy is to navigate the target to the goal(s) with minimal number of movements. Adults should also recognize that they have to perform the series of movements with minimal amount of time. By contrast, given the same instruction children may not fully understand the fact that minimal number of movements should also correspond to the minimal time required to complete the task. This seems consistent with the fact that differences in performance between groups tended to be more apparent in task-solving time than number of movements (**Table 2**). Because it is difficult to clearly differentiate these possibilities in the present study, using modified forms of instructions including nonverbal ones would be promising in future research.

Another point is that the apparent difference in behavior from younger to older participants may largely reflect developmental differences in motor abilities when coping with these touch screen-based tasks. In fact, two of the three measures of performance we examined, task-solving time and first-response latency, could be deemed to reflect the participants' motor skill rather than global/local heuristics. Miyata et al. [16] made detailed analyses of 3- to 4-year-old children's behavior when solving maze problems using the present navigation task and suggested that both planning for each future movement and inhibition of erroneous movements are involved in their performance (see also [21] for evidence in adults). Nevertheless, in the present TSPs, it seems difficult to clearly distinguish between simple motor skills and higher forms of mental strategies such as heuristics. To overcome these limitations, it would be possible to introduce a modified method that places no motor demand such as finger movements. For example, a task using an eye-tracking system that requires distinction of efficient and non-efficient strategies in each TSP could promisingly be applied for young children (see [30]–[32] for use of eye-trackers in the developmental context).

It also seems worthwhile pointing out the differences between the tasks used in the present study and the TSPs commonly used in studies with humans. Specifically, the present tasks had 2 to 3 goals to visit in addition to the starting location of the target. These numbers are relatively small considering the fact that most preceding studies with TSPs involving human participants used tasks having 10 or more nodes [6]–[14]. Also, as mentioned above, the present study used a city-block instead of a Euclidean metric. Consequently, the ways in which the traveling distance can be minimized in the present task was different from those in the TSPs used in many of the preceding studies. Specifically, in most studies on TSPs involving humans [6]–[14], participants were required to connect each node with a straight line and to find the optimal way to shorten the total lengths of the lines. By contrast, in the present task there was a 9×9 matrix within which the target could be moved only in four possible directions, and the participants were required to minimize the total traveling distance. Thus, it may have been more important for the participants in this study to minimize the number of movements in order to visit each goal, rather than to optimize the sequence of multiple goals to visit. This difference should impose considerable limitations on the extent to which the present results can be generalized to humans' performance on Euclidean versions of the problems. The present task using a city-block metric itself has strong points in that it can be applied for different species such as humans and pigeons. Walwyn and Navarro [33] also reported human performance on TSPs that used a city-block metric as well as a Euclidean metric. However, because of the aforementioned limitations, in the present task it might have been difficult to sufficiently bring out higher-order mental capacities that the participants may potentially possess. Thus, in future studies it would be desirable to introduce a commonly used Euclidean metric and TSPs having much larger numbers of nodes.

The empirical approach to TSP in the present study could be further extended from a developmental perspective. It would be plausible that infants and children younger than 3 years show notable development in performance on TSPs, considering that the basic ability to plan for future actions and its precursor to face novel problems is thought to develop during these periods [34]. For developmental stages in which touch screen-based tasks cannot be used, real-world settings such as those in a room or those representing grocery shopping in the town may effectively complement the computerized settings. This seems relevant considering that several studies involving human adults have actually used natural, real-world situations such as shopping downtown or in a supermarket to examine route selection strategies and optimization [35]–[37]. These various approaches should help us to obtain more refined views on the ontogenetic origins of mental processes that guide complex problem solving.
